# Patients’ and partners’ health-related quality of life before and 4 months after coronary artery bypass grafting surgery

**DOI:** 10.1186/1472-6955-12-16

**Published:** 2013-07-08

**Authors:** Patricia Thomson, Catherine A Niven, David F Peck, Jennifer Eaves

**Affiliations:** 1 School of Nursing, Midwifery and Health, BG Bomont Building, University of Stirling, Stirling FK9 4LA, Scotland

**Keywords:** Patients, Partners, Coronary artery bypass grafting, Health-related quality of life

## Abstract

**Background:**

Patients having coronary artery bypass grafting (CABG) often depend on their partners for assistance before and after surgery. Whilst patients’ physical and mental health usually improves after surgery little is known about the partners’ health-related quality of life (HRQoL) in CABG. If the partners’ physical and emotional health is poor this can influence their caregiving role and ability to support the patient. This study aimed: to increase understanding of patients’ and partners’ HRQoL before and after CABG; to explore whether patients’ and partners’ pre-operative socio-demographics and HRQoL predict their own, and also partners’ HRQoL 4 months after CABG.

**Methods:**

This prospective study recruited 84 dyads (patients 84% males, aged 64.5 years; partners 94% females, aged 61.05 years). Patients’ and partners’ perceived health status was assessed using the Short-Form 12 Health Survey. Patients’ physical limitation, angina symptoms and treatment satisfaction were assessed using the Seattle Angina Questionnaire. Partners’ emotional, physical and social functioning was assessed using the Quality of Life of Cardiac Spouses Questionnaire. Data were analysed using hierarchical multiple (logistic) regressions, repeated measures analysis of variance, paired *t* test and Chi square.

**Results:**

Patients most likely to have poorer physical health post-operatively were associated with partners who had poorer pre-operative physical health. Partners most likely to have poorer emotional, physical and social functioning post-operatively were associated with patients who had poorer pre-operative mental health. Patients” and partners’ poorer post-operative HRQoL was also explained by their poorer pre-operative HRQoL.

**Conclusion:**

The partners’ involvement should be considered as part of patients’ pre-operative assessment. Special attention needs be paid to patients’ pre-operative mental health since it is likely to impact on their post-operative mental health and the partner’s emotional, physical and social functioning.

## Background

Coronary heart disease (CHD) is a leading cause of mortality and morbidity both in developed and developing countries [1-5]. A treatment option for patients with advanced atherosclerotic CHD is coronary artery bypass grafting (CABG). In 2009 in the US, more than 416,000 patients underwent bypass surgery [4]; the benefits include relief of angina, improvement in quality of life and increase in life expectancy in high-risk patients [6-11]. Several factors can influence the health-quality of life (HRQoL) of patients after CABG. The main factors identified in large randomised controlled trials are poor pre-operative physical health [7,9,10,12,13], greater severity of angina [7,9,12], and employment status [7,12]. Non-randomised studies suggest increasing age [14-16], male gender [17-20], greater social deprivation [21,22], less education [23], increasing dyspnoea [17,24-26], greater symptom severity/medication use [21,23,24], and previous myocardial infarction [27] contribute significantly to patient’s poorer HRQoL after CABG. The individual’s perception of their health is important since it may differ from objective assessment determined by medical means [28]. Also, depression can be both an indicator and an outcome of CABG [24,29].

Support from the partner (or spouse) has been shown to decrease patient mortality and improve psychosocial recovery after an acute cardiac event [30,31]. Patients often rely on their partner for assistance before and after CABG surgery [32-34]. Several studies have identified positive changes in patient’s physical health and mental health after CABG [21,24,26,35-38], although improvement in mental health is generally slower [24]. Previous studies also suggest a significant interplay between patient’s physical health and mental health [21,24-26,39-42]. Female patients often have poorer outcomes from CABG, compared to males [38,43], and younger age is associated with better post-operative HRQoL [22,42]. In addition, Lie et al. [42] showed that living with a partner and better pre-operative physical health predicted patient’s better physical health after CABG, but only patient’s and not the partner’s HRQoL was assessed.

Surprisingly little is known about partner’s HRQoL in CABG and how this might change from pre- to post-operatively given their crucial role in supporting the patient. Halm et al. [32] found that being a female caregiver and having more caregiver depressive symptoms were associated with negative caregiver outcomes in studies of CABG. Younger age was associated with the caregivers’ poorer physical health. Rantanen et al. (2009) found that patients’ and significant others’ HRQoL 6 months after CABG was explained by their HRQoL 1 month after surgery [33]. However, most CABG studies have focused on patient’s HRQoL and have seldom collected data from patient-partner pairs (i.e. dyads), although such wider data collection has been reported in chronic heart failure or post-myocardial infarction [43-49]. Results from these studies suggest that partners' HRQoL can be worse than the patients’ HRQoL, and their general health and mental health worse compared with age- and sex-matched controls [43,46]. Still little is known about patients’ and partners’ pre- and post-operative HRQoL in CABG or the determinants of their HRQoL after CABG.

Therefore, the aims of this prospective, observational study were to increase understanding of patients’ and partners’ HRQoL before and after CABG and to explore whether patients’ and partners’ pre-operative socio-demographics and HRQoL predict their own, and also partners’ HRQoL 4 months after CABG. Three research questions were identified: 1) what are the changes in patients’ and partners’ HRQoL from before to 4 months after CABG? 2) what are the differences between patients’ and partners’ physical health and mental health before and 4 months after CABG? 3); what are the associations between patients’ and partners’ pre-operative socio-demographics and HRQoL and their own and also the partners’ HRQoL outcomes 4 months after CABG?

## Methods

The patients and partners were seen in the out-patients (OP) clinic before and at home 4 months after CABG. Both their HRQoL were assessed as part of a wider multifactorial, exploratory study. A sample of patient-partner pairs or patient-family pairs were recruited providing: the patient was having a first time elective CABG procedure; was aged 80 years or younger; had moderate to severe coronary artery disease (defined as stenosis greater than 70%, or 50% if left main stem disease) and was married or cohabiting. Partners and close family members (hereafter referred to as partners) were included providing they lived in the same household as the patient and had been identified by them as their main carer and would therefore be sharing the experience of waiting for surgery and recovery at home afterwards. Partners were excluded if they had a history of CHD. Patients and partners were excluded if there were major co-morbidities such as stroke, cancer, renal or liver failure, or communication or psychological limitations likely to affect their ability to consent or participate. Those who met the inclusion criteria were recruited from the cardiac surgery OPs clinic of a regional cardiology centre in Scotland between 2003 and 2004.

### Measures

HRQoL was regarded as a multidimensional construct, to include subjective evaluations of the individual’s physical, mental and social functioning. HRQoL was assessed by measuring patients’ perceived physical health and mental health and the impact of treatment on their physical, mental and social health. Partners’ HRQoL was assessed by measuring their perceived physical health and mental health status, and also their emotional, physical and social functioning, linked to their concerns about the patient and their condition.

#### *The short-form 12 health survey*

Both patients’ and partners’ perceived health status were assessed pre- and post-operatively using the United Kingdom (UK) version of the Medical Outcomes Short-Form 12 Health Survey (SF-12 UK) [50]. The psychometric properties of the SF-12 have been well established and validated in studies of cardiac patients [51,52]. In developing the SF-12 from the original SF-36, the authors reduced its original eight sub-scales to the two summary components (PCS: Physical Component score and MCS: Mental Component Score) [53]. All converted PCS and MCS scores above or below 50 (minimum 0, maximum 100) are above or below the population average [50,51]. In this study, the Cronbach alpha’s for the PCS and MCS were satisfactory with scores ranging between 0.77 and 0.78 for patients, and 0.72 and 0.78 for partners.

#### *Seattle angina questionnaire*

Patients’ pre-operative HRQoL was also assessed using the UK version of the Seattle Angina Questionnaire (SAQ-UK) [54], a disease-specific measure. The SAQ-UK was also used post-operatively when the patients had residual symptoms of angina. The SAQ-UK contains three sub-scales: physical limitation (9 items); angina frequency and perception (7 items); and treatment satisfaction (3 items). Each subscale of the SAQ-UK is rated on a five-point Likert scale from 1 (lowest level) to 5 (highest level of functioning) Scale scores ranged from 0 to 10, obtained by subtracting the lowest possible score and dividing it by the range of the scale and multiplying by 100. The original SAQ has been widely used in studies of cardiac patients [55], when it demonstrated satisfactory validity and reliability [56-58]. The SAQ-UK has comparable validity to the SAQ-US [54], and it has been used in studies of CABG [59]. In the present study, the Cronbach alpha for the SAQ-UK was satisfactory at 0.87.

#### *Quality of life of cardiac spouses questionnaire*

Partners’ HRQoL was also assessed using the Quality of Life of Cardiac Spouses Questionnaire (QL-SP) [60] pre- and post-operatively. It contains two sub-scales: the emotional functional dimension (EFD) or affective component (14 items); and physical and social functional dimension (PSFD) or lifestyle pattern component (12 items). Each subscale consists of items identified as a concern or problem to cardiac partners, rated on a seven-point Likert scale from 1 (all of the time) to 7 (none of the time). The converted EFD scores range from 0 (lowest level) to 98 (highest level of emotional functioning). The PSFD scores range from 0 (lowest level) to 84 (highest level of physical and social functioning). The QL-SP was developed for the spouses of MI patients, when it demonstrated good construct and content validity [60]. It was selected in this study in the absence of a similar measure of HRQoL for the partners of CABG patients although other studies have used a one-item measure of caregivers self reported health status and depressive symptoms [61]. The Cronbach alpha for the QL-SP was satisfactory at 0.82.

#### *Perceived symptom severity*

Patients’ perceived symptom severity was assessed pre-operatively using three separate numerical rating scales (NRS) for: severity of angina; limitation of activities to prevent the onset of angina; and dependence on medication, where 0 represented no limitation/dependence and 10 represented extreme limitation/dependence. They were also used post-operatively when patients had residual symptoms of angina. These 3 items have been previously validated in studies of cardiac patients [62]. The NRS has also been used in pain research [63,64], when it significantly and positively correlated with other measures of pain intensity [65].

#### *Socio-demographics*

Similar socio-demographic data were collected from patients and partners. This included marital status (married/cohabitating or widowed/divorced/separated), years of education, employment status (as recorded by the Office of National Statistics (1998) [66], social deprivation (as defined by Carstairs and Morris’ [67] deprivation categories of 1 (most affluent) to 7 (most deprived)). Patients’ clinical history (i.e. symptoms of angina and breathlessness, Canadian Cardiovascular Society (CCS) grading system, NYHA class, left ventricular ejection fraction and number of diseased vessels) was also obtained from patients’ case notes.

Prior to the main study the above measures were piloted with 10 patients with CHD and their partners. The aim was.to help identify the validity of the questionnaires and practicalities around the study. Results revealed there were no ambiguous questions and it was best to distribute the questionnaires after the patients and partners had seen the cardiac surgeon.

### Procedure

Eighty-four patients and partners or close family members were recruited prior to their visit to the out-patients (OP) clinic. Information about the study and a consent form was posted to potential recruits with the patient’s OP clinic appointment card. Although the patients and partners had the opportunity to discuss participating in the study with each other, we put strategies in place to ensure that they made independent decisions. Written consent was required from each participant prior to scheduling data collection. Upon receiving written consent, verbal consent from each participant was also required, obtained after separating them from each other and asking if they were happy about and willing to take part in the research. It was made clear at this stage that we were interested in recruiting patient-partner pairs and in the event of one member wanting to drop out and in the interests of confidentiality then data collection would continue with the other person, unless they requested otherwise. It was re-iterated that the decision to take part was entirely voluntary and it would not affect the patient’s treatment in anyway. In the event that one partner should die, the decision was taken that data collection with the other partner would stop as it was deemed inappropriate to continue unless the other partner indicated otherwise. Permission to contact patients was requested from the consultant cardiologists and permission to contact partners was sought from their general practitioners.

Over a 4 month period, 208 information packs were sent out, of which 84 consent forms were returned. Overall, 23% of patients and partners agreed to participate. The investigator contacted those that returned the signed consent by telephone and arrangements were made to distribute the questionnaires. The patients and partners were instructed to complete the questionnaires separately from each other and to refrain from discussing their answers. The questionnaires were completed in the OP clinic or returned to the investigators by post. A reminder letter was sent after 2 weeks. For follow-up data collection, the participants were contacted by the researcher and arrangements made to distribute the questionnaires for completion 4 months after the patient’s surgery.

### Ethical considerations

Ethical approval for the study was granted by the University of Stirling and the local National Health Service (NHS) Research and Ethics Committees.

### Statistical analysis

Research Question 1: The chi squared statistic was used for comparison of socio-demographics when data were categorical and the paired *t* test used for continuous data, and to identify changes in HRQoL.

Research Question 2: Repeated measures analysis of variance was used to describe differences between the patients’ and partners’ health status i.e. the physical health component score (PCS) and mental health component score (MCS) before and after CABG (group x time) [68].

Research Question 3: Bivariate relationships were examined using a series of correlation matrixes, using Pearson’s correlation coefficients. First, patients’ post-operative PCS and MCS (outcome variables) and their clinical characteristics (HYHA, CCS), socio-demographics (age, gender, education and occupation) and pre-operative PCS and MCS (independent variables) were examined. Second, patients’ post-operative PCS and MCS (outcome variables) and partners’ socio-demographics (age, gender, education and occupation) and pre-operative PCS and MCS, emotional functional dimension (EFD) and physical and social functional dimension (PSFD) (independent variables) were examined. Third, partners’ post-operative PCS, MCS, EFD and PSFD (outcome variables) and their socio-demographics and pre-operative PCS, MCS, EFD and PSFD (independent variables) were examined. Lastly, partners’ post-operative PCS, MCS, EFD and PSFD (outcome variables) and patient’s socio-demographics, clinical characteristics and pre-operative PCS and MCS (independent variables) were examined. Only the independent variables that significantly correlated with the dependent (outcome) variables at 0.30 or above were tested in separate hierarchical multiple (logistic) regression models.

The independent variables were entered hierarchically into the multiple (logistic) regression models in a predetermined order. For example, patients’ (or partners’) pre-operative PCS and MCS (SF-12) were entered in step 1; physical limitation, angina frequency and severity, and dependence on medication (SAQ-UK) and perceived symptom severity (NRS) were entered in step 2 (and EFD and PSFD (QL-SP) for partners). Socio-demographics and clinical characteristics were entered in step 3, as necessary, to achieve the best model fit with up to 4 independent variables. Hierarchical multiple (logistic) regression was used because most dependent (outcome) variables were bimodal in distribution, dichotomised using the median split method [68]. For example, the EFD was encoded as 1 for the group with the lowest score (35.00-64.00) and 0 for the group with normal or above scores (66.00-98.00).

Power calculations for the hierarchical multiple (logistic) regression showed a sample size of 80 (160 patients and partners) was sufficient to detect medium effect sizes (f^2^ = 0.15, 80% power), with up to 4 predictor variables in the models [69]. The dependent variables were: patients’ and partners’ post-operative PCS and MCS scores, and partners’ post-operative EFD, PSFD. All analyses were performed using SPSS version 19.0 for Windows and *P* < 0.05 indicated statistical significance.

## Results

### Personal characteristics

Eighty-four patients and partners or close family members participated in the study. There were 79 patient-partner pairs and five patient-family pairs, including two daughters, a sister, a son and brother. Socio-demographics, clinical history and attendance at cardiac rehabilitation are presented in Table 1. There were 80 patient-partner pairs remaining at 4 months follow-up. Two patients died whilst on the waiting list for CABG, 1 patient died within 24 hours of surgery and 1 patient had his surgery postponed until after he had lost weight and stopped smoking; all their partners withdrew from the study.

**Table 1:** Summary of socio-demographics, clinical history and rehabilitation

**Characteristics**	**Patients**	**Partners**	***p***
Mean age in years (median, range)	64.54 (65.00,40-80)	61.05 (63.01,24-82)	<0.001
Gender			
Males	71 (85%)	11 (13%)	<0.001
Females	13 (15%)	73 (87%)	
Employment:			
Employed	17 (20%)	31 (37%)	0.030
Unemployed	7 (8%)	11 (13%)	
Retired	60 (71%)	42 (50%)	
Occupation:			
Professional – intermediate	26 (31%)	11 (13%)	0.046
Skilled non manual –manual	19 (23%)	20 (24%)	
Partly skilled – unskilled	39 (46%)	53 (63%)	
Mean years of education (median, range)	11.57 (10.00, 9–21)	11.04 (10.00, 9–20)	0.742
Social deprivation:			-
Depcat 1 - 2	24 (28%)	-	-
Depcat 3 – 5	41 (49%)	-	
Depcat 6 – 7	19 (23%)	-	
Hypertension	53 (63 %)	7 (8%)	<0.001
Diabetes mellitus	19 (23%)	2 (2%)	<0.001
Angina	78 (93%)	-	
Mean age onset of angina (median, range)	54.8 (60.00, 40–79)	-	
Breathlessness	46 (55%)	-	
Myocardial infarction (MI)	32 (38%)	-	
Mean age of first MI (median, range)	52.7 (60.50, 32–75)		
Number of first MI	27 (32%)	-	
Canadian Cardiovascular Society (CCS)			
CCS 1 – 2	42 (50%)	-	
CCS 3 – 4	47 (56%)	-	
Missing or no chest pain	6 (7%)	-	
New York Heart Association (NYHA)			
Class 1 - 2	32 (38%)	-	
Class 3 - 4	36 (43%)	-	
Missing	5 (6%)	-	
Left venticular ejection fraction			
> 50%	55 (65%)	-	
30 – 49% (moderate impairment)	20 (24%)	-	
< 29% (severe impairment)	2 (3%)	-	
Missing	7 (8%)	-	
Number of diseased vessels			
Single-vessel disease	7 (8%)	-	
Two-vessel disease	28 (34%)	-	
Three-vessels	43 (51%)	-	
Missing	6 (7%)	-	
Mean waiting time for surgery in days	63.17	-	
Length of hospital stay days (median, range)	7 (4–21)	-	
Attendance at cardiac rehabilitation	50 (62%)	2 (2%)	<0.001

*Depcat*, social deprivation categories; p < 0.05.

### Changes in health-related quality of life

Patients’ PCS and MCS scores improved significantly from pre- to post-operatively (Table 2). The pre-operative SAQ scores for the entire patient sample were: physical limitation (mean 48.76, SD 24.47); angina frequency and perception (mean 31.25, SD 19.23); and treatment satisfaction (mean 84.52, SD 14.06). The pre- and post-operative scores for the 8 patients with residual symptoms of angina are presented in Table 2. Significant improvements were noted for physical limitation and angina frequency, but not treatment satisfaction. The numerical rating scale (NRS) scores for these patients also changed significantly from pre- to post-operatively: severity of angina (t = −4.072, df 7, p = 0.005), limitation of activities to prevent onset of angina (t = −2.539, df 7, p = 0.011) and dependence on medication (t = −2.226, df 7, p = 0.025), indicating that they had gained some benefits from surgery. In contrast, the partners’ PCS and MCS scores did not change significantly from pre- to post-operatively, although significant improvement was noted in their emotional functional dimension (EFD) and physical and social functional dimension (PSFD) (QL-SP) (Table 2).

**Table 2:** Changes in patients and partners pre – and post-operative health-related quality of life

**Variable**	**Patients pre-op**	**Patients post-op**			**Partners pre-op**	**Partners post-op**		
	**Mean (SD)**	**Mean (SD)**	**t**	***p***	**Mean (SD)**	**Mean (SD)**	**t**	***p***
SF12 (n = 80)								
PCS	30.45 (8.64)	41.47 (10.94)	−10.87	0.001	46.92 (10.92)	45.94 (11.13)	0.88	0.382
MCS	44.17 (11.50)	48.19 (11.63)	−3.05	0.003	45.81 (11.34)	47.48 (11.48)	−0.90	0.370
SAQ (n = 8)								
PLS	29.46 (17.78)	66.07 (23.30)*	- 4.17	0.004		-		
AFP	18.28 (15.22)	52.65 (28.67)*	- 2.65	0.333		-		
TS	71.87 (10.85)	65.50 (27.81)*	1.94	0.380		-		
QLSP (n = 80)								
EFD	-	-			64.00 (15.25)	73.86 (15.40)	−5.48	0.001
PSFD	-	-			57.09 (12.26)	64.87 (10.60)	−5.88	0.001

*SF12* Short-form 12 health survey, *PCS* physical component score, *MCS* mental component score, *SAQ* Seattle angina questionnaire, *PL* physical limitation score, *AFP* angina frequency and perception, *TS* treatment satisfaction, *QLSP* Quality of life cardiac spouse, *EFD* emotion functional dimension, *PSFD* physical and social functional dimension, * n = 8.

### Differences in perceived health status

**Figure 1:**
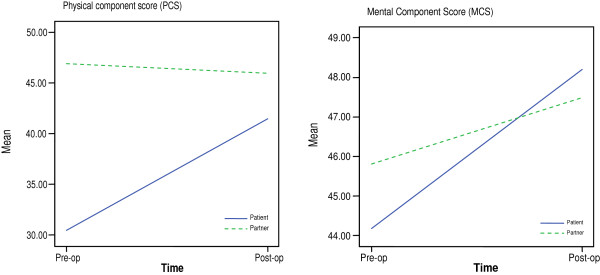
Differences in patient’s and partner’s physical health and mental health related quality of life pre-and post-operatively.

Results of the repeated measures ANOVA revealed a significant time effect (interaction) between the patients’ and partners’ PCS pre- and post-operatively (F (1, 79) = 67.77, p 0.001), effect size of 0.462 (Eta^2^) (Figure 1). About 46% of the variance in scores was accounted for by differences between the groups and by differences over the two time periods. No significant time effect was noted between the patients’ and partners’ MCS pre- and post-operatively (F (1, 79) = 3.30, p = 0.073) (Figure 1), indicating no differences between the groups and by differences over the two time periods.

### Patients’ post-operative physical and mental health and pre-operative socio-demographics and HRQoL

Table 3 shows the hierarchical multiple (logistic) regression results for patients’ post-operative PCS and their pre-operative factors. In step 2 of the model, the factors that were significantly associated with a greater likelihood of being a member of group 1 (poorer PCS) were: patients’ pre-operative PCS and MCS, which explained 50% of the variance. In Table 4 the hierarchical multiple (logistic) regression results for the patients’ post-operative MCS indicate that their pre-operative PCS and MCS were significantly associated with a greater likelihood of them being a member of group 1 (poorer MCS), explaining 38% of the variance.

**Table 3:** Multiple logistic regression predicting patient’s post-operative physical health (PCS)

**Patient’s post-op physical health (PCS) (dependent variable)**							**95.0% C.I**
	**Beta**	**S.E.**	**Wald**	***d***	***p***	**Lower**	**Upper**
**Patient pre-op** (independent) variables							
*Step 1: chi square = 29.075, -2 Log Likelihood = 76.776, R = .305, Nagelkerke R*^*2*^ *= .415*							
PCS	-.131	.037	12.016	1	0.001**	.816	.945
MCS	-.075	.027	7.616	1	0.006*	.880	.979
*Step 2: chi square =7.864, -2 Log Likelihood =68.911, R = .370, Nagelkerke R*^*2*^ *= .504*							
PCS	-.214	.006	10.403	1	0.001**	.709	.919
MCS	-.110	.036	9.182	1	0.002*	.834	.962
Physical limitation	.007	.022	.086	1	0.769	.964	1.051
Angina freq/severity	.053	.028	3.541	1	0.060	.998	1.114
Constant	9.311	2.511	13.753	1	<0.001	-	-
**Partner pre-op** (independent) variables							
*Step 1: chi square = 12.425, -2 Log Likelihood = 93.425, R = .144, Nagelkerke R*^*2*^ *= .19*							
PCS	-.059	.029	5.743	1	0.007*	.883	.988
MCS	-.038	.025	2.335	1	0.126	.917	1.011
*Step 2: chi square = .002, -2 Log Likelihood = 3.423, R = .144, Nagelkerke R*^*2*^ *= .106*							
PCS	-.069	.030	5.321	1	0.021*	.880	.990
MCS	-.039	.032	1.499	1	0.221	.904	1.024
EFD	.001	.023	.002	1	0.968	.956	1.048
*Step3: chi square = 4.515, -2 Log Likelihood = 88.909, R = .191, Nagelkerke R*^*2*^ *= .260*							
PCS	-.069	.033	3.739	1	0.050*	.878	1.001
MCS	-.067	.036	3.393	1	0.065	.872	1.004
EFD	.028	.029	1.077	1	0.316	.974	1.086
Social deprivation	−1.899	1.006	3.559	1	0.059	.021	1.077
Constant	6.547	1.971	11.029	1	<0.001	-	-

*PCS* physical component score, *MCS* mental component score, *EFD* emotion functioning dimension, *angina freq/severity* angina frequency and severity, *post-op* post-operative, * p < 0.005; ** p < 0.001.

**Table 4:** Multiple logistic regression predicting patient’s post-operative mental health (MCS)

**Patient’s post-op mental health (MCS) (dependent variable)**							**95.0% C.I**
	**Beta**	**S.E.**	**Wald**	***d***	***p***	**Lower**	**Upper**
**Patient pre-op** (independent) variables							
*Step 1: chi square = 24.744, -2 Log Likelihood = 86.109, R = .266, Nagelkerke R*^*2*^ *= .355*							
PCS	-.083	.034	5.875	1	0.015*	.861	.984
MCS	-.087	.025	11.90	1	0.001**	.872	.963
*Step 2: chi square =1.842,-2 Log Likelihood =84.267, R = .324, Nagelkerke R*^*2*^ *= .377*							
PCS	-.108	.056	3.642	1	0.056*	.804	1.003
MCS	-.104	.033	10.065	1	0.002*	.845	.961
Physical limitation	-.005	.022	.047	1	0.769	.953	1.039
Angina freq/severity	.033	.024	1.862	1	0.172	.986	1.084
Constant	11.132	3.045	13.365	1	<0.001	-	-
**Partner pre-op** (independent) variables							
*Step 1: chi square = 5.794, -2 Log Likelihood = 105.060, R = .075, Nagelkerke R = .100*							
PCS	-.052	.023	5.314	1	0.021*	.910	.998
MCS	-.014	.022	4.791	1	0.514	.821	.908
*Step 2: chi square = 12 .129,-2 Log Likelihood = 92.186, R = .208, Nagelkerke R*^*2*^ *= .268*							
PCS	-.032	.931	1.474	1	0.225	.924	1.028
MCS	-.020	.024	.731	1	0.398	.936	1.026
Gender	−2.559	1.106	5.355	1	0.021*	.008	.653
Social deprivation	−1.421	.676	4.440	1	0.035*	.062	.900
Constant	.946	1.333	.504	1	0.478	-	-

*PCS* physical component score, *MCS* mental component score, *EFD* emotion functioning dimension, *angina freq/severity* angina frequency and severity, *post-op* post-operative, * p < 0.005; ** p < 0.001.

Table 3 also shows the hierarchical multiple (logistic) regression results for patients’ post- operative PCS and partners’ pre-operative factors. In step 3 of the model, the factor significantly associated with a greater likelihood of patients being a member of group 1 (poorer PCS) was: the partners’ pre-operative PCS which explained about 27% of the variance. In Table 4 the hierarchical multiple (logistic) regression results for the patients’ post-operative MCS indicate the partners’ (female) gender and greater social deprivation were associated with a greater likelihood of patients’ being a member of group 1 (poorer MCS), but the model failed to reach statistical significance.

### Partners’ post-operative physical and mental health and pre-operative socio-demographics and HRQoL

Table 5 shows the hierarchical multiple (logistic) regression results for partners’ post-operative PCS and their pre-operative factors. In step 3 of the model, the factor significantly associated with a greater likelihood of partners’ being a member of group 1 (poorer PCS) was: their pre-operative PCS, which explained 55% of the variance. In Table 6 the hierarchical multiple (logistic) regression results for the partners’ post-operative MCS indicate their pre-operative PCS and MCS were significantly associated with a greater likelihood of them being a member of group 1 (poorer MCS), explaining 39% of the variance.

**Table 5:** Multiple logistic regression predicting partner’s post-operative physical health (PCS)

**Partner’s post-op physical health (PCS) (dependent variable)**							**95.0% C.I**
	**Beta**	**S.E.**	**Wald**	***d***	***p***	**Lower**	**Upper**
**Partner pre-op** (independent) variables							
*Step 1: chi square = 27.372, -2 Log Likelihood = 83.53, R = 290, Nagelkerke R*^*2*^ *= .386*							
PCS	-.137	.035	15.229	1	0.001**	.814	.934
MCS	-.077	.025	.069	1	0.792	.945	1.044
*Step 2: chi square =1.524, -2 Log Likelihood =82.011, R = .303, Nagelkerke R*^*2*^ *= .404*							
PCS	-.152	.038	15.649	1	0.001**	.797	.926
MCS	-.038	.037	1.050	1	0.002*	.895	1.035
EFD	.035	.030	1.379	1	0.306	.977	1.097
Step 3: *chi square = 4.384, -2 Log Likelihood = 64.527, R = .403, Nagelkerke R*^*2*^ *= .550*							
PCS	-.153	.040	14.520	1	0.001**	.793	.938
MCS	-.049	.038	1.664	1	0.197	.884	1.026
EFD	.049	.033	2.255	1	0.133	.984	1.119
Social deprivation	-.939	.923	1.035	1	0.309	.064	2.388
Constant	7.239	2.005	13.032	1	<0.001	-	-
**Patient pre-op** (independent) variables							
*Step 1: chi square = 6.876, -2 Log Likelihood = 104.027, R = .082, Nagelkerke R*^*2*^ *= .110*							
PCS	-.039	.028	1.896	1	0.169	.910	1.017
MCS	-.039	.021	3.421	1	0.064	.923	1.002
*Step 2: chi square = .325, -2 Log Likelihood = 103.702, R = .086, Nagelkerke R*^*2*^ *= .115*							
PCS	-.056	.044	1.591	1	0.207	.867	1.032
MCS	-.045	.025	3.278	1	0.070	.911	1.004
Physical limitation	.010	.018	.320	1	0.571	.975	1.048
Angina freq/severity	-.003	.017	.028	1	0.866	.964	1.032
Constant	3.271	1.411	5.375	1	0.020	-	-

*PCS* physical component score, *MCS* mental component score, *EFD* emotion functional dimension, *angina freq/severity* angina frequency and severity, *PSFD* physical and social functional dimension, *post-op* post-operative, * p < 0.005; ** p < 0.001.

**Table 6:** Multiple logistic regression predicting partner’s post-operative mental health (MCS)

**Partner’s post-op mental health (MCS) (dependent variable)**							**95.0% C.I**
	**Beta**	**S.E.**	**Wald**	***d***	***p***	**Lower**	**Upper**
**Partner pre-op** (independent) variables							
*Step 1: chi square = 17.895, -2 Log Likelihood = 93.008, R = 200, Nagelkerke R*^*2*^ *= .267*							
PCS	-.056	.025	4.860	1	0.027*	.900	.994
MCS	-.067	.025	.6.975	1	0.008*	.890	983
*Step 2: chi square =17.897, -2 Log Likelihood =93.006, R = .200, Nagelkerke R*^*2*^ *= .267*							
PCS	-.055	.027	4.363	1	0.037*	.898	.997
MCS	-.066	.032	4.169	1	0.041*	.879	.997
EFD	-.001	.024	.002	1	0.963	.952	1.048
Step 3: *chi square = 0.999, -2 Log Likelihood = 83.268, R = .373, Nagelkerke R*^*2*^ *= .389*							
PCS	-.057	.027	4.432	1	0.035*	.896	.996
MCS	-.006	.032	4.090	1	0.043*	.879	.998
EFD	-.007	.032	.045	1	0.781	.945	1.078
PSFD	.009	.034	.077	1	0.781	.945	1.078
Constant	5.639	1.683	14.389	1	0.001	-	-
**Patient pre-op** (independent) variables							
*Step 1: chi square = 6.645, -2 Log Likelihood = 103.683, R = .086, Nagelkerke R*^*2*^ *= .115*							
PCS	-.020	.026	.568	1	0.451	.931	1.032
*Step 2: chi square = 6.645,-2 Log Likelihood = 103.683, R = .086, Nagelkerke R*^*2*^ *= .115*							
PCS	.060	.042	2.017	1	0.156	.978	1.152
Physical limitation	-.039	.018	4.791	1	0.029*	.928	.996
Angina freq/severity	.001	.017	.003	1	0.956	.968	1.034
*Step 3: chi square = 4.634,-2 Log Likelihood = 99.049, R = .138, Nagelkerke R*^*2*^ *= .184*							
PCS	.062	.044	1.969	1	0.161	.976	1.161
Physical limitation	-.042	.019	5.089	1	0.024*	.924	.994
Angina freq/severity	.000	.017	.000	1	0.983	.967	1.034
Age	.057	.028	4.258	1	0.039*	1.003	1.118
Constant	−3.559	1.983	3.219	1	0.073	-	-

*PCS* physical component score, *MCS* mental component score, *EFD* emotion functional dimension, *angina freq/severity* angina frequency and severity, *PSFD* physical and social functional dimension, *post-op* post-operative, * p < 0.005.

Table 5 also shows the hierarchical multiple (logistic) regression results for partners’ post-operative PCS and patients’ pre-operative factors. There were no patient pre-operative variables that were significant predictors of partners’ post-operative physical health. In Table 6 the hierarchical multiple (logistic) regression results for the partners’ post-operative MCS indicate that patients’ pre-operative greater physical limitation and increasing age were associated, but the model failed to reach statistical significance.

### Partners’ post-operative emotional, physical and social functional dimensions and pre-operative socio-demographics and HRQoL

Table 7 shows the hierarchical multiple (logistic) regression results for partners’ post-operative emotional functional dimension (EFD) and their pre-operative factors. In step 2 of the model, the factors that were significantly associated with a greater likelihood of being a member of group 1 (poorer EFD) were: partners’ pre-operative EFD, PCS and MCS, which explained about 54% of the variance. In Table 8 the hierarchical multiple (logistic) regression results for the partners’ post-operative physical and social functional dimension (PSFD) indicate their pre-operative PSFD was significantly associated with a greater likelihood of being a member of group 1 (poorer PSFD), explaining about 38% of the variance.

**Table 7:** Multiple logistic regression predicting partner’s post-operative emotion functional dimension (EFD)

**Partner’s post-op emotion functional dimension (EFD) (dependent variable)**							**95.0% C.I**
	**Beta**	**S.E.**	**Wald**	***d***	***p***	**Lower**	**Upper**
**Partner pre-op** (independent) variables							
*Step 1: chi square = 24.552, -2 Log Likelihood = 74.822, R = .264, Nagelkerke R*^*2*^ *= .372*							
EFD	-.122	.036	11.378	1	0.001**	.824	.950
PSFD	.049	.039	1.589	1	0.207	.973	1.134
*Step 2: chi square = 13.529,-2 Log Likelihood = 60.940, R = .381, Nagelkerke R*^*2*^ *= .536*							
EFD	-.095	.045	4.508	1	0.034*	.833	.993
PSFD	.086	.047	3.381	1	0.066	.994	1.194
PCS	-.092	.033	7.808	1	0.005*	.855	.973
MCS	-.082	.042	3.900	1	0.048*	.849	.999
Constant	9.311	2.511	13.753	1	<0.001	-	-
**Patient pre-op** (independent) variables							
*Step 1: chi square = 21.665, -2 Log Likelihood = 77.708, R = .237, Nagelkerke R = .334*							
PCS	-.110	.045	6.075	1	0.014*	.821	.978
MCS	-.080	.027	8.679	1	0.003*	.876	.974
*Step 2: chi square = 0.522, -2 Log Likelihood = 77.187, R = .242, Nagelkerke R*^*2*^ *= .341*							
PCS	-.079	.061	1.724	1	0.189	.820	1.048
MCS	-.068	.031	4.759	1	0.029*	.878	.993
Physical limitation	-.014	.023	0.387	1	0.534	.942	1.032
Angina freq/severity	-.002	.020	0.010	1	0.920	.961	1.037
Constant	5.042	1.910	6.971	1	0.008	-	-

*EFD* Emotion functional dimension, *PSFD* physical & social functional dimension, *PCS* physical component score, *MCS* mental component score, *angina freq/severity* angina frequency and severity, *post-op* post-operative * p < 0.005; ** p < 0.001.

**Table 8:** Multiple logistic regression predicting partner’s post-operative physical and social functional dimension (PSFD)

**Partner’s post-op physical and social functional dimension (PSFD) (dependent variable)**							**95.0% C.I**
	**Beta**	**S.E.**	**Wald**	***d***	***p***	**Lower**	**Upper**
**Partner pre-op** (independent) variables							
*Step 1: chi square = 21.782, -2 Log Likelihood = 84.069, R = .238, Nagelkerke R*^*2*^ *= .325*							
EFD	-.021	.028	0.576	1	0.448	.927	1.034
PSFD	-.083	.038	4.783	1	0.029*	.845	.991
*Step 2: chi square = 3.948,-2 Log Likelihood = 80.120, R = .275, Nagelkerke R*^*2*^ *= .375*							
EFD	.013	.034	.145	1	0.703	.948	1.082
PSFD	-.087	.040	4.618	1	0.032*	.847	.992
PCS	-.022	.027	.650	1	0.420	.928	1.032
MCS	-.058	.033	3.015	1	0.082	.884	1.007
Constant	7.185	1.920	13.998	1	0.001**	-	-
**Patient pre-op** (independent) variables							
*Step 1: chi square = 20.953, -2 Log Likelihood = 84.897, R = .230, Nagelkerke R = .314*							
PCS	-.062	.036	3.079	1	11.646	.876	1.007
MCS	-.089	.026	11.646	1	0.001**	.876	.974
*Step 2: chi square = 3.501, -2 Log Likelihood = 81.385, R = .263, Nagelkerke R*^*2*^ *= .359*							
PCS	-.017	.052	0.103	1	0.748	.887	1.090
MCS	-.080	.030	6.961	1	0.008*	.870	.980
Physical limitation	-.038	.022	2.904	1	0.088	.922	1.006
Angina freq/severity	.026	.021	1.466	1	0.226	.984	1.070
Constant	4.357	1.680	6.728	1	0.009	-	-

*EFD* Emotion functional dimension, *PSFD* physical and social functional dimension, *PCS* physical component score, *MCS* mental component score, *angina freq/severity* angina frequency and severity, *post-op* post-operative * p < 0.005; ** p < 0.001.

Table 7 also shows the hierarchical multiple (logistic) regression results for partners’ post-operative emotional functional dimension (EFD) and patients’ pre-operative factors. In step 2 of the model, the factor significantly associated with a greater likelihood of the partners’ being a member of group 1 (poorer EFD) was the patients’ pre-operative MCS which explained 34% of the variance. In Table 8 the hierarchical multiple (logistic) regression results for partners’ post-operative physical and social functional dimension (PSFD) indicate that patients’ pre-operative MCS was significantly associated with a greater likelihood of the partners’ being a member of group 1 (poorer PSFD), explaining about 36% of the variance.

In summary, results indicate that the patients most likely to have poorer post-operative PCS (but not mental health) were associated with partners’ who had poorer pre-operative PCS. Patients’ poorer post-operative PCS was also explained by their own poorer pre-operative PCS and MCS. The results indicate that partners most likely to have poorer post-operative emotional functioning (emotional functional dimension, EFD) and physical and social functioning (physical and social functional dimension, PSFD) were associated with patients’ who had poorer pre-operative MCS. In addition, partners’ poorer post-operative EFD was also explained by their poorer pre-operative EFD, PCS and MCS; and their poorer post-operative PSFD, PCS and MCS were explained by their poorer pre-operative PSFD, PCS and MCS.

## Discussion

This study aimed to increase understanding of patients’ and partners’ HRQoL before and after CABG and to explore whether their pre-operative socio-demographics and HRQoL predict their own, and also partners’ HRQoL 4 months after CABG. An important finding was that the patients most likely to have poorer physical health post-operatively were associated with partners’ who also had poorer pre-operative physical health. Earlier research on patients’ and significant others’ HRQoL at baseline (1 month after CABG) found that it influenced their later HRQoL 6 and 12 months after surgery [33]. However, no studies have specifically examined the associations between partners’ pre-operative HRQoL and patients’ post-operative physical health, which makes the comparison of results difficult. However, our finding is important because if partners’ health is poor to begin with this may be detrimental to patients’ health and recovery after CABG. Partners’ pre-operative physical health might therefore be considered as part of patients’ pre-operative assessment since they may need practical support and advice from health care professionals, in addition to the social support provided by their own social network [33,61,70]. Research with couples has also highlighted that when people marry and share the same house, income and social network this can confer shared health risks and benefits [71]. This suggests a need for health education and prevention targeted at both patients and partners.

In the study, there was no indication in that there was an association between patients who had poorer post-operative mental health with partners’ poorer pre-operative HRQoL. No previous studies have specifically examined the associations between patients’ post-operative mental health and partners’ pre-operative HRQoL dimensions. Previous researchers have however identified that pre-operative MCS scores are independent predictors of lower post-operative MCS scores in the CABG patient population (26,40,62). In the study, the bivariate analysis revealed that patients’ post-operative mental health was significantly correlated with partners’ gender and social deprivation i.e. being female and having greater social deprivation were related to patients’ poorer mental health after CABG. Earlier studies have similarly found significant associations between patients’ HRQoL and partner’s female gender [33]. Other researchers have identified a significant relationship between CABG patients’ mental health and social deprivation [21,22]. Given that partners share the same household and environment, it is not surprising that partners’ social deprivation was negatively associated with the study patients’ post-operative mental health.

In the study we found that patients’ poorer post-operative physical health was also explained by their poorer pre-operative physical and mental health. Previous studies have identified that patients’ pre-operative measurement of physical health and mental health predicts their HRQoL after CABG [7,9,10,12,13,24,26,40],[41]. Our study results concur with earlier research, and also highlight the interrelatedness of the physical and mental HRQoL components and so the importance of patient’s biopsychosocial assessment. Patient’s age and gender did not contribute significantly in their post-operative HRQoL, which is consistent with other short-term studies post CABG [33]. There have been some contradictory findings though with other studies identifying gender and age as associated with poorer physical recovery after CABG [14,16,18-20]. The differences in these findings may be due to the study designs, the populations studied and the HRQoL dimensions measured. Previous CABG studies have often used the 8 sub-scales of the SF-36 [21,26,39,40], which makes comparison of research difficult. Previous research suggests patients’ NYHA class correlates with mental health after CABG i.e. higher the NYHA class is associated poorer mental health [17,24-26,42], but we found no such association, and this may be due to the different times to follow-up.

In the study there was no indication that the partners most likely to have poorer post-operative mental health were associated with patients with poorer pre-operative HRQoL. It is not entirely clear why this should be, but it may be related to the generic nature of the instrument used i.e. the SF-12. In the bivariate analysis, partners’ post-operative mental health was significantly associated with the patients’ pre-operative physical limitation and age i.e. the patients’ greater physical limitation and increasing age was associated with the partners’ poorer post-operative mental health. These findings are broadly consistent with previous research [72] that has found worse patient proxy health ratings and less personal mastery to be associated with greater partner (caregiver) depressive symptoms. Worse patient proxy health ratings were also associated with worse caregiver perceived physical health [72].

In contrast, results indicate that partners’ post-operative emotional functional dimension (EFD) and physical and social functional dimension (PSFD) (QL-SP) were significantly associated with the patients’ pre-operative mental health i.e. there was a greater likelihood of partners’ having poorer post-operative emotional, social and physical functioning if patients had poorer pre-operative mental health. These findings highlight the importance of addressing patients’ pre-operative mental health to help improve partners’ HRQoL after CABG, and so the capacity of partners to assist patients in the recovery period after surgery. In general, there is in a scarcity of empirical evidence on partner HRQoL outcomes in CABG. No studies have focused specifically on the association between partners’ post- operative HRQoL and patients’ pre-operative mental health.

It was not an unexpected finding that the partners’ greater likelihood of having poorer post-operative emotional, physical and social functioning (EFD, PSFD) was explained by their pre-operative EFD and PSFD scores. In previous research, Ebbensen et al. [60] found that partners’ EFD and PSFD scores were associated with their earlier EFD and PSFD in partners (spouses) of post MI patients [60]. Partners’ greater likelihood of having poorer post-operative physical health was also explained by their poorer pre-operative physical health; their poorer post-operative mental health was also explained by their poorer pre-operative physical and mental health. In previous research, the partner’s (caregiver’s) depressive symptoms were associated with negative caregiver outcomes in CABG [72].

Results from the repeated measures ANOVA indicate significant differences between the patients’ and partners’ perceived physical health (PCS) before and after CABG. Whilst the patients’ physical health improved significantly after CABG there was no corresponding improvement in the partners’ physical health. This may be because patients’ had physical limitation and angina symptoms before CABG so there was greater potential for improvement in physical health after surgery. It might also be related to the lower mean age of the partners, who were on average, 3.5 years younger than the patients. Results further indicate there were no significant differences between the patients’ and partners’ mental health before and after CABG. The patients’ mental health improved significantly from pre- to post-operatively, but there was no corresponding improvement in the partners’ mental health. Similarly, Rantenen et al. [33] found that patients had a lower level of mental function than their significant others after CABG. Other investigators in heart failure have found that patients with CHD can have poorer physical health but not mental health than their spouses [73]. In our study the partners’ pre-operative mental health was not as low as the patients before CABG and so there was less room for improvement afterwards. Another factor that might explain the similarity between the patients’ and partners’ MCS scores is the shared experience i.e. they might both have worries and concerns about the patient’s forthcoming surgery and recovery at home afterwards [74]. Halm et al. highlight these concerns are likely to be greater when patients have been in poorer health and when the partners (or spouses) themselves have been in poorer health [74]. We also found that although patients’ mental health improved significantly from pre – to post-operatively this change was modest, compared to improvement in physical health. Other investigators have also found this relatively slow improvement in mental functioning, compared with physical functioning [21,24], which is perhaps not unexpected.

The partners’ emotional functional dimension (EFD) and physical and social functional dimension (PSFD) (QL-SP) both improved significantly from pre- to post-operatively. We found the QL-SP to be a responsive and valid measure in the study despite it being developed originally for the partners (spouses) of MI patients [60]. When tested the measure demonstrated good internal consistency. However, use of the QL-SP and responsiveness with the partners of CABG patients needs to be further tested in research. Overall, the patients’ and partners’ post-operative physical and mental health remained below the population average, compared the Scottish Health Survey [75] and despite some improvement the partners’ had a sub-optimal level of emotional, physical and social functioning as measured by the QL-SP [60] 4 months after CABG.

The main strength of the study was its longitudinal design. We collected as far as possible parallel data from CABG patients and their partners. Furthermore, we recruited married and cohabitating partners, which is significant because cohabitating couples have not always been considered in research. We considered patients and partners in CABG, whilst previous research has focused mostly on chronic heart failure or post MI [43,44].

### Implications

First, to improve CABG patients’ physical health after surgery it may be necessary to consider the partners’ physical health as part of pre-operative assessment. This may help to predict those patients most at risk of through lack of assistance and support. Second, to improve partners’ post-operative HRQoL and their potential to support patients it may be necessary to consider patients’ pre-operative mental health. Third, further research is needed to confirm or refute our findings. This is important because current emphasis is on partners as collaborators in self management and the role of family functioning in reducing hospital readmissions [61]. Moreover, partner support has been shown to facilitate successful patient recovery and adjustment following coronary revascularisation, but there is limited research examining the partner HRQoL before and after CABG. By implication, we need to consider the impact of CABG on the partners of patients having surgery and how this might influence patient outcomes. This could be considered as part of the patients’ discharge planning and cardiac rehabilitation. Fourth, although we did not present information on social support, previous research has identified the importance of partner (spousal) support before and after CABG [33,34,70,76]. Fifth, the results of this study lend support to the design of an intervention that targets both CABG patients and partners to help improve HRQoL outcomes of patients after surgery, and the feasibility such an approach as part of recovery and cardiac rehabilitation.

### Limitations

There are limitations to this study. The response rate was relatively low but this is not unusual in studies that seek to recruit both patients and partners [77,78]. It may also be affected by staff within the OP clinic who recruited the participants. No data were collected on multiple morbidities. Patients and partners who did not participate in the study may have greater morbidity and complex health needs. Data were collected about nine years ago but it may still be relevant today. Current research still highlights the impact of an acute cardiac event on the partners of patients (30). Less frequently studied is the HRQoL of patients having CABG and their partners. This study was unique in collecting, as far as possible, parallel data from patients and their partners before and after CABG. This research is important in extending understanding related to partners (caregivers) research and expands upon the previously reported literature. We reported the findings of logistic regression which puts new light on the partners’ status. Our analysis examined between pairs (dyadic) analysis. Future researchers may want to explore within pairs analysis to determine whether the patients’ and partners’ pre-operative variables predict their own, and also the partner’s HRQoL after CABG, using longitudinal dyadic regression for distinguishable dyads [79].

## Conclusions

The patients most likely to have poorer physical health post-operatively were those with partners who also had poorer pre-operative physical health. The partners most likely to have poorer emotional functioning (emotional functional dimension, EFD) and poorer physical and social functioning (physical functional dimension, PSFD) post-operatively were associated with patients who had poorer pre-operative mental health. Furthermore, patients’ poorer post-operative physical and mental HRQoL was explained by their own poorer pre-operative physical and mental health. The partners’ poorer post-operative HRQoL was also explained by their own poorer pre-operative physical and mental HRQoL and poorer emotional, physical and social functioning. The partners’ involvement should be considered as part of patients’ pre-operative assessment. Special attention needs be given to the patients’ pre-operative mental health since it is likely to impact on their own post-operative mental health, and also the partner’s emotional, physical and social health and functioning after CABG.

## Abbreviations

CABG: Coronary artery bypass grafting; CCS: Canadian cardiovascular society; CHD: Coronary heart disease; EFD: Emotion functional dimension; HRQoL: Health related quality of life; MCS: Mental component score; NRS: Numerical rating scales; NYHA: New York heart association class; OP: Out-patient; PCS: Physical component score; PSFD: Physical and social functional dimension; QL-SP: Quality of life of cardiac spouses questionnaire; SAQ-UK: Seattle angina questionnaire United Kingdom version; SAQ: Seattle angina questionnaire; SF-12 UK: Short-form 12 health survey United Kingdom version; US: United States.

## Competing interests

The authors declare that they have no competing interests.

## Authors’ contributions

PT designed the study, collected data, conducted the statistical analysis and wrote the manuscript. KN and DFP contributed to the study design, they provided critical feedback on the study and statistical analysis, and inputted to the draft of this manuscript. JE contributed to the literature review and statistical analysis. All authors have read and approved the final manuscript.

## Pre-publication history

The pre-publication history for this paper can be accessed here:

http://www.biomedcentral.com/1472-6955/12/16/prepub
